# Laser-Powered Homogeneous Pyrolysis (LPHP) of Lignin Dispersed into Gas Phase

**DOI:** 10.3390/molecules30102215

**Published:** 2025-05-19

**Authors:** Mohamad Barekati-Goudarzi, Lavrent Khachatryan, Rubik Asatryan, Dorin Boldor, Bert C. Lynn

**Affiliations:** 1 Department of Chemistry, Louisiana State University, Baton Rouge, LA 70803, USA; barekatimohamad@gmail.com (M.B.-G.); lkhach1@lsu.edu (L.K.); 2 Department of Chemical and Biological Engineering, University at Buffalo, The State University of New York, Buffalo, NY 14260, USA; 3 Department of Biological & Agricultural Engineering, Louisiana State University and Louisiana State University AgCenter, Baton Rouge, LA 70803, USA; dboldor@agcenter.lsu.edu; 4 Department of Chemistry, University of Kentucky, Lexington, KY 40506, USA; bclynn2@uky.edu

**Keywords:** PAH, lignin pyrolysis, laser reactor, depolymerization, soot, radicals

## Abstract

The gas-phase delivery of lignin into the hot zone of cw-CO_2_ laser-powered homogeneous pyrolysis (LPHP) reactor under “wall-less” conditions led to the breakdown of lignin macromolecules into neutral oligomers and paramagnetic fragments deposited onto the reactor cell walls. The formation of PAHs was observed during the defragmentation of lignin, accelerated with increased laser power. Remarkably, no phenolic compounds were detected among lignin fragments—intermediate radicals and neutral oligomers. It is concluded that the PAH and soot-like conjugated particulates are formed in the hot zone of the LPHP reactor, resembling the high-temperature combustion processes. The key role of the resonantly stabilized radicals in the formation of low-molecular-weight PAHs is outlined. An alternative pathway is proposed for the generation of PAH involving the formation of cyclopentadienyl radical precursors (CPDa) that are adsorbed onto or trapped within lignin macromolecules.

## 1. Introduction

Understanding and monitoring the production of bio-oils by fast biomass pyrolysis is an efficient way to optimize the involved chemical processes and improve their subsequent refinement. To bypass intractable secondary processes during the solid-phase pyrolysis of lignin, we have recently introduced a straightforward approach involving the initial gasification of lignin macromolecules. Lignin dissolved in a solution of acetone/water (9:1 ratio) was pulverized into a gas phase in a contactless, tubular reactor [1,2]. The overall goal of the experiments was to limit the side effects typically accompanying the pyrolysis of solid-state lignin in conventional reactors. Depending on the delivery modes, two different types of reactors were utilized: (1) Continuous Droplet Evaporation (CDE) and (2) Constant Output Atomization (CA) reactors. Lignin was transported through the isothermal zone by either a syringe pump (CDE reactor) or a TSI atomizer (CA reactor). The results revealed new features for the degradation of hydrolytic lignin (HL) dispersed into the gas phase compared to those in the conventional batch reactors. In particular, the formation of intermediate oligomers in both reactors was shown to occur, involving only trace amounts of phenolic compounds [1,2].

Some preliminary results on the pyrolysis of lignin in the gas phase using a “wall-less”, cw-IR CO_2_ laser-powered homogeneous pyrolysis (LPHP) non-isothermal reactor were briefly reported elsewhere [3,4]. The temperature distribution in the LPHP reactor was monitored by thermocouple measurements validated by the method of “chemical thermometer” and COMSOL Multiphysics simulations [3,4]. Large differences were found between the gas-phase depolymerization of lignin in the LPHP reactor and conventional reactors, yet much similarity was seen with CDE and CA reactors. The gas-phase delivery of lignin into the hot zone of the LPHP reactor under “wall-less” conditions led to the breakdown of lignin into neutral and paramagnetic fragments that deposited onto the cell walls [3]. Because of the steep/narrow temperature zone in the center of the LPHP reactor, these fragments readily leave the hot zone and quench/stabilize in the cold areas of the reactor, primarily on the cold walls.

No phenolics were detected in the LPHP reactor; instead, the formation of polycyclic aromatic hydrocarbons (PAHs) was observed, and the yields of PAHs accelerated with increasing laser power. Note that the accumulation of PAHs in the conventional reactors, as detailed in [5], tends to increase with temperature. However, a critical decrease is observed in the yields of PAHs containing two fused aromatic rings. This insight is essential for regulating the composition of PAH mixtures based on temperature variations.

Understanding the underlying chemistry of PAHs formation observed virtually in any biomass conversion and combustion process, refs. [6,7] presents a significant challenge [8]. While PAHs are typically perceived as toxic and unwanted byproducts of biomass pyrolysis, they could serve as biomass-derived precursors for valuable carbon-based materials, including carbon fibers [9], films [10], hydrogels [11], etc. Additionally, the inclusion of small quantities of low-molecular-weight PAHs as additives in diesel fuel enhances the physicochemical properties of diesel surrogate fuel blends, leading to improvements in both energy density and viscosity [12,13]. Note that a diesel surrogate blend is a simplified fuel mixture tailored to replicate diesel’s combustion and physical properties [14]. Generally, diesel fuel is composed of C_10_∼C_24_ hydrocarbons, including 50–65% alkanes (mostly n-alkanes), 20–30% cycloalkanes, and 10–30% aromatics [15]. For instance, the addition of methyl naphthalene in a concentration of 13 to 21% to 3, 5, and 7 components of a diesel surrogate fuel blend, for instance, accurately reproduces actual engine combustion characteristics [14].

The goal of this work is to explore the applicability of the laser-powered pyrolysis of lignin in well-known combustion-related processes, leading to the formation of low-molecular-weight (LMW) aromatics and “younger” soot macromolecules within the hot zone of the LPHP reactor. In addition to the homogeneous reaction channels, an alternative “heterogeneous” pathway for PAH formation at elevated temperatures is also proposed, involving reactions of cyclopentadienyl (CPDa) radicals adsorbed onto HL macromolecules.

## 2. Results and Discussion

**Figure 1:**
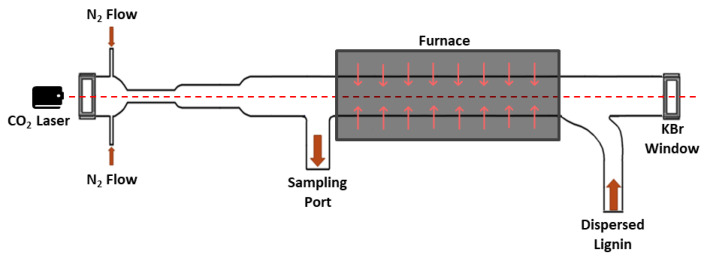
Schematic of the IR countercurrent LPHP reactor.

Due to the complexity of the direct temperature measurements, computational numerical modeling was undertaken to simulate and identify the temperature distribution in the IR countercurrent reactor, as shown in Figure 1.

COMSOL Multiphysics (v5.3a, COMSOL Inc, Burlington, MA, USA), a finite element analysis (FEA) software package, was used to solve the ordinary differential equations (ODEs) and partial differential equations (PDEs) involved in the laser light absorption and transition into heat transfer in the fluid and fluid dynamic models, using a procedure modified in our previous work [4]. The initial phase of computational temperature measurements, along with the experimental validation for a similar system, can be found elsewhere [3].

### 2.1. Temperature Distribution in LPHP Countercurrent Reactor

**Figure 2:**
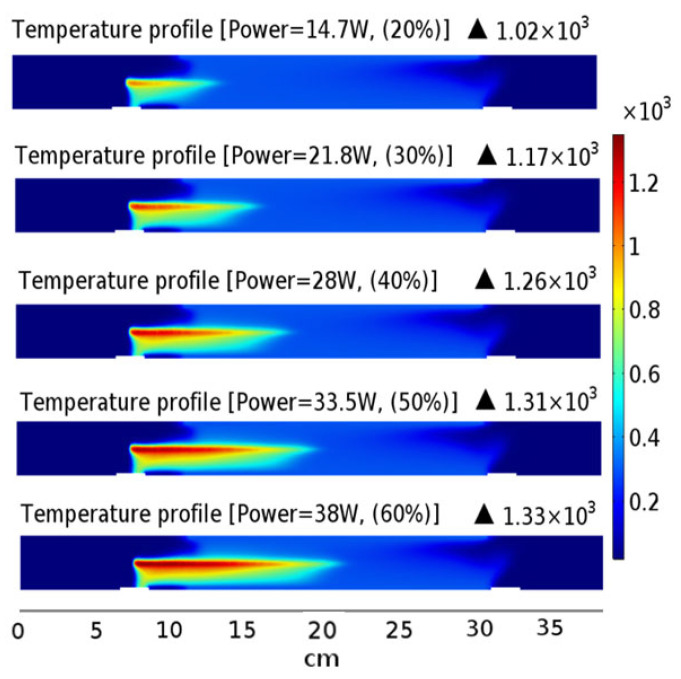
COMSOL prediction of the “hot zone” temperature shown on a cross-sectional plane along axial direction (Z coordinate) at 14.7, 21.8, 28, 33.5, 38 W laser power with 2% SF_6_ in N_2_ gas mixture under atmospheric conditions.

The temperature profiles inside the LPHP reactor based on COMSOL Multiphysics simulation are mapped in Figure 2.

For these calculations, three types of mesh (coarse, normal, and fine mesh) were studied with respect to the efficiency of simulation and accuracy of the results. As expected, by elevating the laser power, the temperature of the hot zone increases and propagates over a longer distance at the onset of absorption. The laser beam has a 2.5 mm diameter where the maximum radial temperature is calculated.

Considering a volume with temperature above 400 °C as the reacting zone for each laser power, the calculated residence time of the lignin in the hot zone, for a 3 L/min carrier gas flowrate, can be evaluated to range from 40 ms to 160 ms, corresponding to the 14.7 W to 38 W laser power, respectively. The high maximum temperature shown in Figure 2 for each laser power can compensate for such a short residence time to achieve reasonable conversion rates. Note, the TSI 3076 Constant Output atomizer generates extremely fine aerosols with a number-mean droplet diameter of 0.30 µm from the lignin solution, ideal to be rapidly heated to the temperature achieved in the hot zone. Moreover, the steep temperature profile achieved in this reactor and fast cooling of pyrolysis products provide great conditions for obtaining the primary products or preserving intermediates emerging from lignin pyrolysis.

### 2.2. Condensable Products from Countercurrent LPHP Reactor

**Figure 3:**
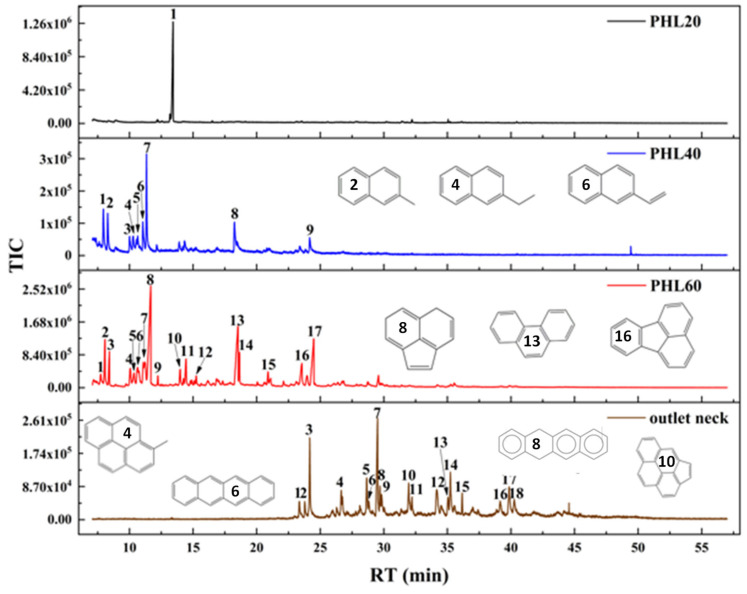
GC-MS chromatograms of the products of the homogeneous pyrolysis of lignin in a counter-current LPHP reactor (Figure 1) at various laser powers and locations in the reactor. HL mass transfer rate ~2.5 µg/s, residence time 100 ms, outside reactor temperature 300 °C. The product identifications are shown in Tables S1–S4 (Supplementary Materials). The laser power assignments PHL 20, 40, and 60 stand for pyrolyzed hydrolytic lignin (PHL) at laser power of 20% (14.7 W), 40% (28 W), and 60% (38 W). #1 compound at PHL20 is guaiacol, which most probably formed from vinyl guaiacol; the latter one is an intrinsic compound in HL. TIC refers to Total Ion Count, and RT denotes retention time in minutes.

The GC-MS (Agilent 6890Nbruker) results from the countercurrent LPHP reactor are represented in Figure 3 (the product identifications are provided in Tables S1–S4, Supplementary Materials) at different laser powers from 14.7 to 38 W. The products condensed in the cold trap were extracted with acetone at the end of the experiment and analyzed via GC-MS.

Surprisingly, no phenolics (phenols, guaiacols, syringols, etc.) were detected in noticeable amounts. Different fused aromatic rings containing PAHs were detected with increasing laser power at ascending order of aromatic rings in PAHs, Figure 3, (Tables S1–S4, Supplementary Materials). Notably, the PAHs were not detected below 22 W laser power.

**Figure 4:**
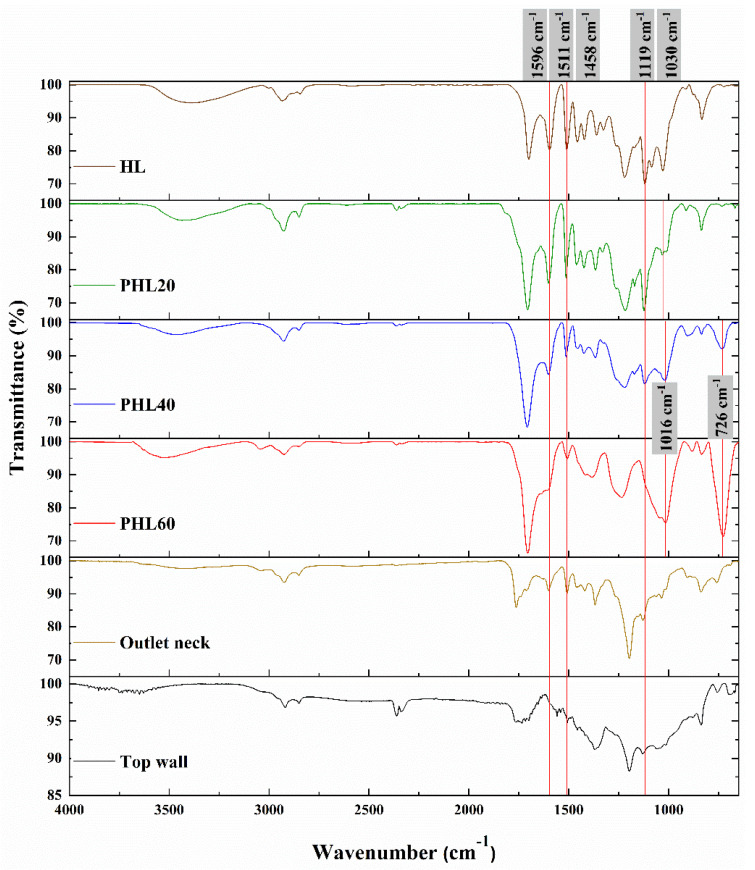
FTIR spectra of the condensed products in the trap from lignin homogeneous pyrolysis in the LPHP reactor at laser power of 20% (14.7 W), 40% (28 W), and 60% (38 W), and the deposited products on the outlet neck and top wall. PHL—pyrolyzed HL. The laser power assignments PHL 20, 40, and 60 stand for pyrolyzed hydrolytic lignin (PHL) at laser power of 20% (14.7W), 40% (28 W), and 60% (38 W).

The formation of PAHs was also identified by FTIR (Bruker Tensor 27, Ettlingen, Germany) analysis of trapped products from the sampling port of the LPHP reactor; the frequency at 726 cm^−1^ is a characteristic vibration mode for the C–H wags in an aromatic ring in fused ring systems, as shown in Figure 4, at PHL 40 and PHL 60; refer to the abbreviations in Figure 4 caption. These peaks, however, are missing in the FTIR spectra of deposited products on the outlet neck and top wall of the reactor, as shown in Figure 4. 

In other words, the emergence of the strong peak at 726 cm^−1^ (spectra at PHL 40 and PHL 60, Figure 4), due to the C–H wags for an aromatic ring with 3, 4, and 5 adjacent hydrogen atoms, indicates the formation of fused ring systems, such as substituted naphthalene, anthracene, or phenanthrene. These compounds were abundant among products trapped during pyrolysis at high laser power.

A detailed peak identification of the FTIR spectra is presented in Table 1. It is obvious that as the energy of the laser increases, the major aromatic structural units in the lignin macromolecules, labeled as the most characteristic bands at 1596 and 1511 cm^−1^, and 1119 and 1030 cm^−1^ for C=C and C–H vibration in the skeletal aromatic ring, break down, as shown in Figure 4.

Demethoxylation constitutes another phenomenon that is intensified when the temperature is increased, as observed from the FTIR spectra. Bands at 1458, 1085, and 1030 cm^−1^ gradually disappeared, showing the complete removal of the methoxy functional groups from the substituted aromatic rings in both trapped and deposited products. Similar behavior was reported during conventional pyrolytic char formation of lignin [18]. These results, along with the results indicating the formation of soot particulates detected by FTIR from the outlet neck and top wall of the LPHP reactor (Figure 4), are consonant with literature data on conventional pyrolysis of lignin and cellulose at high temperatures [18,19].

GPC measurements described below were performed to further analyze the content and behavior of trapped products not detectable by GC-MS.

### 2.3. Molecular Weight Distribution of the Lignin Homogeneous Pyrolysis Products

#### 2.3.1. GPC Analysis

**Figure 5:**
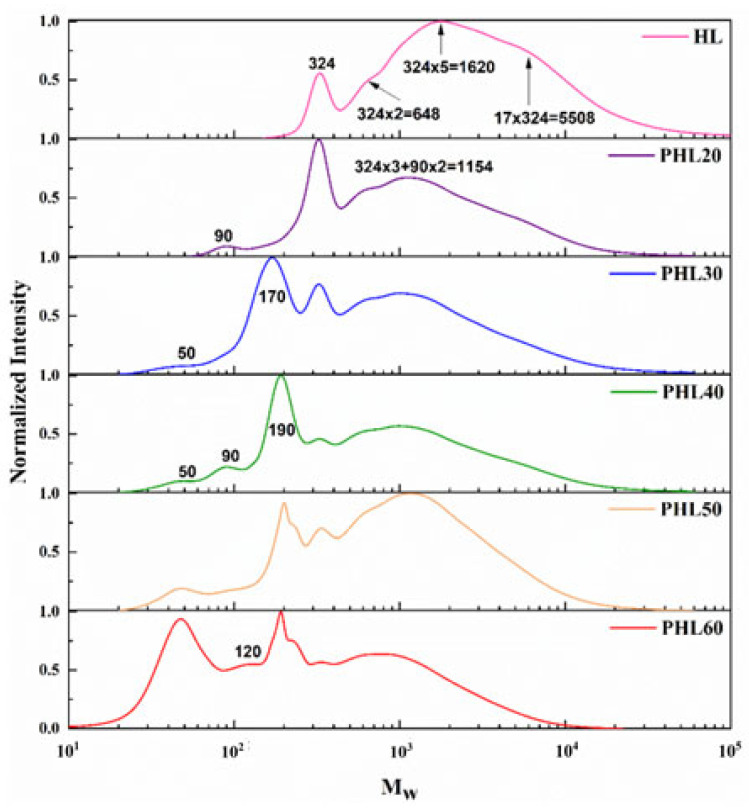
GPC spectra for initial HL vs. products from HL pyrolysis (collected in the trap) at various laser powers in LPHP reactor: 20% (14.7W), 30% (21.8W), 40% (28 W), 50% (33.5W), 60% (38 W). The laser power assignments PHL 20, 30, 40, 50, 60 stand for pyrolyzed hydrolytic lignin (PHL) at laser power of 20% (14.7W), 30% (21.8W), 40% (28 W), 50% (33.5W), 60% (38 W).

To better understand the nature of the hydrolytic lignin gas-phase depolymerization process during homogeneous pyrolysis in the LPHP reactor, gel permeation chromatography (GPC) was employed to examine the molecular weight distribution of the products. The results are shown in Figure 5 for initial lignin (HL) in comparison with depolymerized lignin (PHL) at various laser powers.

These GPC spectra show the various fractions of the hydrolytic lignin and how they change as the lignin structure is deconstructed during depolymerization. As the temperature of the pyrolysis increases, the summits of these fractions move toward lower molecular weights while the new peaks, indicating the new fragments, are also formed and subsequently intensified. Even though the GPC is the most common method to determine molecular weight distribution among many researchers, this technique has a major drawback. Obtained by this method, Mw values are relative to other compounds such as polystyrene. Thus, one cannot draw a definite conclusion based on the values reported in Table 2. The weight-average molecular weights (M_w_), the number-average molecular weights (M_n_), the polydispersity indexes (PDI) of the initial lignin, and the generated products after pyrolysis are provided for comparison purposes.

The molecular weight values of the intact lignin sample, M_n_ and M_w_ of 1397 and 5591 g/mol, respectively, are significantly higher than those for the rest of the pyrolyzed sample, indicating more efficiency of the homogeneous pyrolysis in the gas phase in the depolymerization of hydrolytic lignin. It is also evident that by increasing the laser power, these values reduce gradually as a result of the increased pyrolysis temperature.

Furthermore, the polydispersity index (PDI) of the intact lignin is lower than that of all the pyrolyzed samples except the PHL20 sample, which was pyrolyzed at 20% of the maximum laser power. Collectively, these results suggest that the lignin macromolecules are broken down into smaller fragments and spread over a broader range of molecular weight values, as shown in Figure 5.

This observation contrasts with previous reports in the literature regarding the effect of temperature on the molecular weight of pyrolytic lignin [20,21,22]. Based on the reviewed studies, an increasing trend in the molecular weight distribution, as determined by GPC, was observed with higher pyrolysis temperatures for kraft lignin, organosolv lignin, spruce organosolv lignin, wheat straw organosolv lignin, and milled pine wood lignin. Ragauskas and Ben, for instance, observed that the molecular weights are increased with increasing pyrolysis temperature for both fast and slow pyrolysis oils produced from pine wood [20]. Kersten et al. explained this phenomenon in terms of the interplay of chemistry and mass transport [21]. They reported heavier bio-oil recovery during pyrolysis, regardless of the lignin type, by increasing temperature due to the higher escape rate of heavier components from the reacting region and the low partial pressure.

In fact, lignin macromolecules undergo fragmentation during homogeneous gas-phase processes in the LPHP reactor, seemingly indicating that products in other reactors are involved in the secondary repolymerization reactions, which increase the Mw of the final products. It is pertinent to mention that both ESI–MS and GPC results on the primary pyrolysis products of organosolv lignin at temperatures between 360 and 700 °C show a negligible effect of the pyrolysis temperature on the molar weight distribution using an original vacuum screen heater [22].

#### 2.3.2. The Radical Character of the Decomposition of Lignin in LPHP Reactors

**Figure 6:**
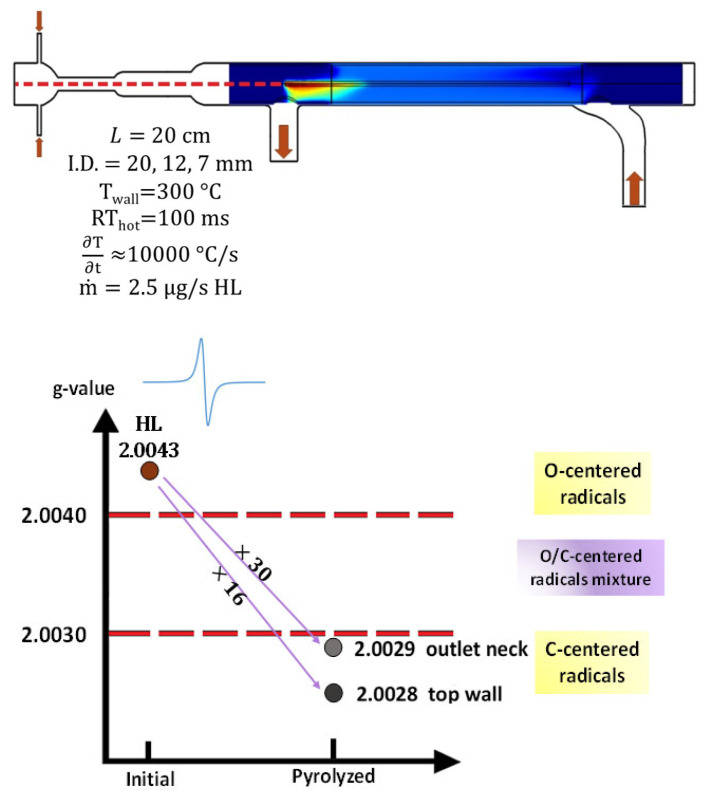
Brown deposits (g-value of 2.0029) on the reactor outlet neck with x16 higher EPR signal intensity than that in initial HL. Black deposits (g-value of 2.0028) on the reactor top wall with x30 higher intensity (at 28W of laser power) than in HL. The g-values 2.0030 and 2.0040 on the Y-axis are arbitrary marker values for C-, O-centered radicals, respectively, as well as a mixture of these radicals between these values.

A dramatic new environment for the pyrolysis of HL in the LPHP reactor, excluding direct contact between volatiles and residue chars, leads to the principal simplification of the primary decomposition processes of the lignin macromolecules. As a result, the major fraction of the pyrolysis products (~80%, w) represents a mixture of the initial intact HL and its depolymerized fragments—oligomers and oligomer radicals—as described in the current work and mentioned in refs [2,3]. The converted lignin (~10–15%, w) deposited on the cold areas, outside of the reactor, shows a high content of radicals, Figure 6. The low g values of the detected radicals from the neck of the countercurrent reactor equal to 2.0029 and 2.0028 confirms the removal of most of the lignin functional groups containing polar groups by the formation of “naked” soot-like conjugated substances; a notable change in the g values of the intrinsic radicals (2.0043, initial lignin, inset, Figure 6) occurs.

Generally, the presence of the macro-radicals provides strong binding conditions to develop the initial clusters of PAHs [23].

### 2.4. Formation Mechanism of PAHs from HL Gas-Phase Pyrolysis in LPHP Reactor

#### 2.4.1. Combustion-Related Homogeneous Channels for Formation of PAHs:

The formation of PAHs and soot particulates (Figure 3 and Figure 4) from lignin gas-phase decomposition in the LPHP reactor can be explained by the traditional multizone soot particulate formation mechanism in hydrocarbon flames. In the pre-flame zone, the fuel or combustible precursors are vaporized at temperatures ranging from ambient up to about 1200 °C. In the flame zone, where the temperature exceeds 1200 °C, the fuel species undergo complete combustion; this zone is associated with the formation of soot and other organic pollutants. The molecular growth of PAHs and soot inception occurs in the post-flame zone with decreased temperatures ranging from 1200 to 600 °C. Below 600 °C, the soot inception/formation rate is exceedingly low.

The possible mechanisms relevant to the growth of aromatics and PAHs/soot particulate formation have been actively disputed in the literature over the last three decades. The current primary focus is the generation of the first few aromatic rings out of small aliphatic compounds. This is perceived by many to be a rate-limiting stage in the reaction sequence leading to larger aromatics, and it is commonly accepted that the formation of the first aromatic ring is the kinetic bottleneck in the soot formation chain [23,24,25]. Subsequent growth of PAHs from a single aromatic species can proceed via a repetitive sequence of hydrogen abstraction and acetylene addition reactions (seminal HACA mechanism) [23,24,25].

Various mechanisms of the formation of PAHs at high temperatures are also largely discussed in the literature [5,26,27,28,29]. In particular, an aryl–aryl combination [30], phenyl or methyl addition/cyclization [31], and others (see detailed discussion in Supplementary Materials, Section S4) have also been reported.

The formation of naphthalene [3] and its derivatives directly observed in the LPHP reactor, as shown in Figure 3, suggests the importance of its formation channels (reaction (S1) and reaction (S3) in Supplementary Materials, Section S4).

2C_5_H_5_ —> C_10_H_8_ + 2H (1)

This involves a key, cyclopentadienyl, C_5_H_5_ (CPD) [32,33], and naphthalenyl radicals [34].

The formation of CPD radicals from pyrolysis/oxidation reactions of numerous lignin contracted models (aromatics) such as catechol, hydroquinone, and phenol has been shown in our early publications [35,36,37], and more recently [38], it has been suggested to occur during the pyrolysis of *p*-coumaryl alcohol at elevated temperatures, >700 °C, as shown in Figure S3. The expulsion of CO from phenoxy rings is typically considered to lead to the formation of cyclopentadienyl and hydroxy-cyclopentadienyl radicals, particularly from the pyrolysis of catechol, hydroquinone [39], guaiacol [40], and para-coumaryl alcohol [38]. Further recombination of such radicals forms naphthalene, indene, hydroxy indene, and other derivatives, as experimentally established in isothermal reactors [35,36,37].

The role of the resonance-stabilized cyclopentadienyl and other aromatic radicals in soot formation has also been indirectly demonstrated recently during the pyrolysis/oxidative pyrolysis of 1-methylnaphthalene at ~1100 °C in a two-section tubular combustion reactor [41]. It should be noted that the CPD radicals are more stable than phenoxy radicals at such high temperatures (>700–1000 °C) [36]. Accordingly, the ratio [CPD]/[PhO] = 2.0 at 700 °C was suggested to increase to 88.4 at 1000 °C in a numerical modeling study using a known scheme of phenol pyrolysis reactions analyzed in detail in the same publication [36]. The formation of derivatized CPD radicals from lignin key models and monomers, particularly para-coumaryl alcohol at elevated temperatures, has been justified theoretically by Asatryan et al. based on the first-principles analysis of reaction mechanisms [42] (detailed in the Supplementary Materials, Figure S4).

**Scheme 1:**
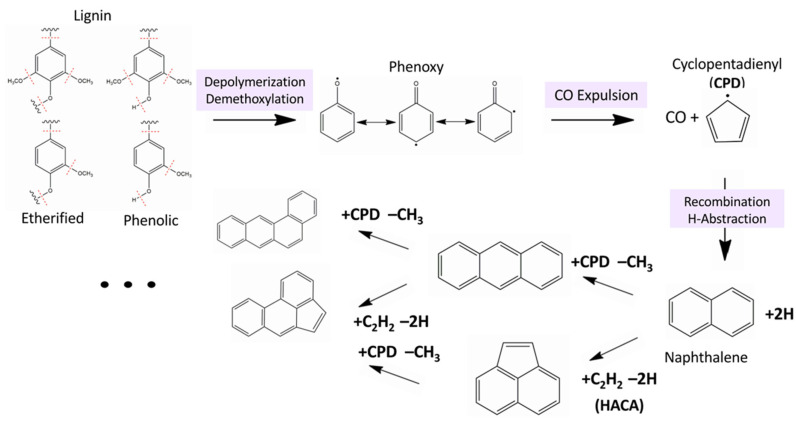
Mechanistic homogeneous pathway of PAHs formation during high-temperature gas-phase pyrolysis of lignin.

Thus, due to the flash heating at elevated temperatures and intense de-functionalization of lignin macromolecule (intermediate oligomers), the formation and further reactions of CPD radicals can occur as described in Scheme 1 (and Figure S5). In other words, the naphthalene formation initiates the generation and growth of myriads of low-molecular-weight (LMW) PAHs (Figure 3), both benzenoid- (phenanthrene, pyrene, etc.) and cyclopentane-based fused (acenaphthylene, fluoranthene, etc.) PAH structures, through both HACA mechanisms and/or addition of the new cyclopentadienyl radicals, [24,43,44,45], as summarized in Figure S5.

A detailed analysis of the formation of soot particulates from LPHP using ESI MS and LDI MS techniques is underway, while similar results from the CA reactor on the detection of soot particulates up to 2000–2500 g/mol after the pyrolysis of lignin dispersed into the gas phase in isothermal conditions have already been reported [2].

#### 2.4.2. A “Heterogenous” Mechanism for Formation of PAHs

As it was mentioned above, we did not detect any phenolics from co-current and counter-current LPHP reactors in the entire range of laser power from 20 to 38W. Therefore, it could be concluded that phenolics do not form in these conditions (decomposition reactions, particularly CO expulsion from a phenoxyl-type radical being dominant over hydrogen abstraction reactions, which lead to phenolics), and they rapidly convert into PAHs and soot particulates at elevated temperatures, as soon as they are formed (≥900–1000 °C, Figure 2), as discussed in Section 2.4.1 above.

An alternative possibility is a heterogeneous formation of PAHs that also involves CPD radicals. The phenoxyl radicals released from the destruction of lignin macromolecules (Scheme 1) stay trapped between polar lignin sub-units, and further expulsion of CO from the phenoxy unit leads to the formation of adsorbed CPD radicals (CPDa) on the lignin macromolecule. Consequently, the surface reactions of CPDa, due to their known higher mobility (see Supplementary Materials, Section S6), can produce PAHs via a scenario described in Scheme 1 (Figure S5).

One could assume a similar mobility of the adsorbed and stabilized CPDa radicals on the surface of the HL macromolecule (as discussed in Supplementary Materials, Section S6), perhaps to a lesser extent due to the polar nature of the lignin branches; the formation of naphthalene, according to reaction (S1), can be envisioned at elevated temperatures.

**Figure 7:**
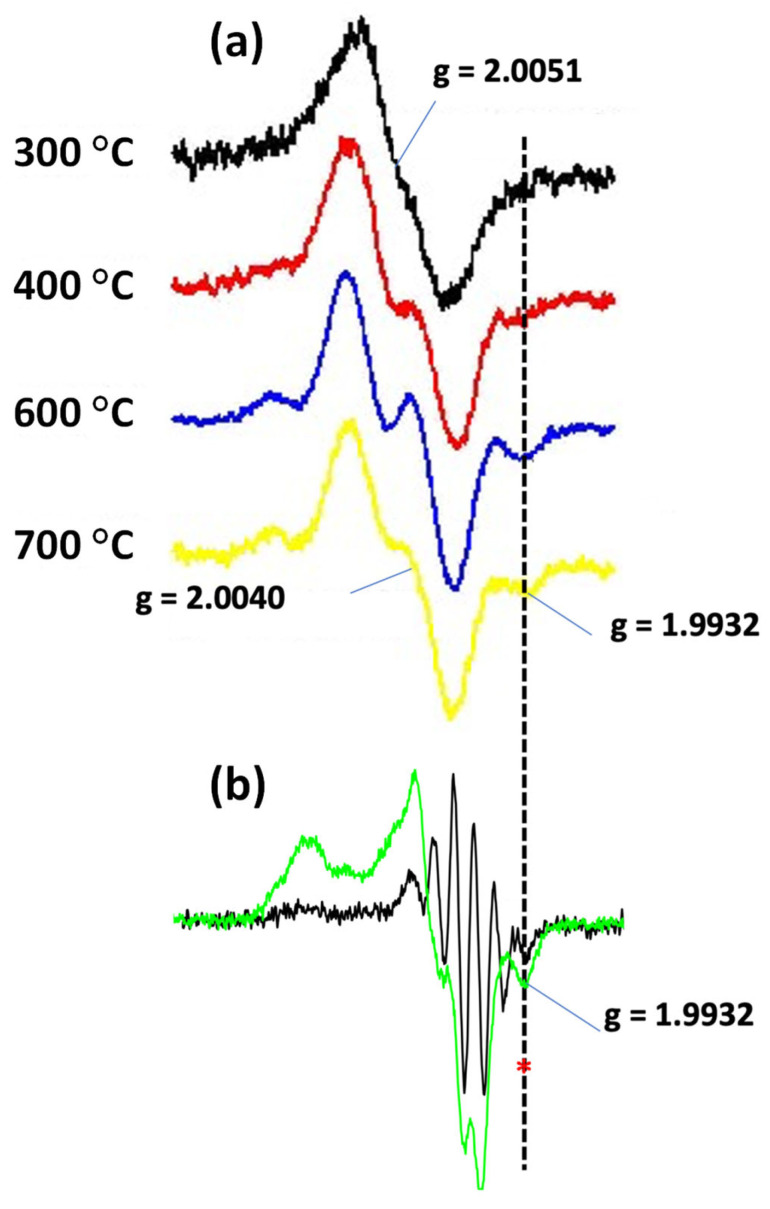
(**a**) Top four EPR spectra were detected at room temperature from lignin pyrolysis in pyroprobe at 300 °C (black), 400 °C (red), 600 °C (blue), and 700 °C (yellow) set temperatures. Radicals were accumulated in the Cambridge filter. (**b**) The two spectra below present a reference EPR spectrum from vacuum pyrolysis of tricarbonylcyclopentadienylmanganese (black line, reaction (S1) in Figure S3) overlayed with the radicals EPR spectrum detected from the vacuum pyrolysis of *p*-coumaryl alcohol at elevated temperatures (>700 °C); both spectra were detected at liquid N2 temperature. The red asterisk shows the reference line from the CPD radical at g = 1.9932.

Evidence on formation of the immobilized CPD radicals into the lignin lattice: Evidence of embedded CPD radicals (CPDa) on the lignin matrix has been provided in our early pyroprobe fast fractional pyrolysis experiments on lignin [46]. The products released at different temperatures in the fast flow of nitrogen gas were collected on a Cambridge filter (CF) at room temperature (Figure S6) and subjected to EPR analysis, as shown in Figure 7a. A broad, structureless EPR signal with g = 2.0051 observed at 300 °C gradually evolves into a well-resolved, complex EPR spectrum as the pyrolysis temperature increases to 600 °C. The EPR spectra seem to be a superposition of EPR signals of several paramagnetic species. A further increase in the temperature to 700 °C leads to the distortion of the well-resolved spectrum at 600 °C with g = 2.0061 at the center to generate another signal with a lower g = 2.0039. A trend can be traced in conversion into the single line at temperatures higher than 700 °C. A benchmark EPR line at g = 1.9932 from the top spectra in Figure 7a is compared with the reference EPR spectrum of pure CPD generated from the vacuum pyrolysis of tricarbonylcyclopentadienylmanganese (Figure S3 [38,47]), as shown by the black line in Figure 7b. The line at g = 1.9932 of the CPD radical does not overlay with any other EPR lines of an organic radical generated from the vacuum pyrolysis of a number of lignin model compounds to provide evidence of the presence of CPD radicals [36]. Importantly, it exactly fits with the same line for the radicals generated from the vacuum pyrolysis of *p*-coumaryl alcohol (green line in Figure 7b) and identified as traces of CPD after the annihilation of the radical mixture [38], as shown in Figure S3. Therefore, the immobilization of CPD radicals on a lignin matrix appears feasible, and the subsequent reactions of these adsorbed radicals, as discussed above, may be highly relevant.

In summary, the reaction sequences presented in Scheme 1 (Figure S5) up to the generation of LMW PAHs may occur both homogeneously in the gas phase and heterogeneously on the surface of the macromolecules of HL.

## 3. Materials and Methods

### 3.1. Materials

Hydrolytic lignin (HL) is produced by isolating lignin from lignocellulosic biomass during hydrolysis [48]. It primarily consists of complex aromatic polymers derived from phenylpropanoid monomers. More specifically, HL is rich in lignin structures consisting of guaiacyl (G), syringyl (S), and p-hydroxyphenyl (H) units. These units are interconnected through various linkages, such as β-*O*-4, β–β, and α-*O*-4 bonds, thus forming a highly cross-linked three-dimensional network.

HL was purchased from Sigma-Aldrich (St. Louis, MO, USA) (a currently discontinued product, 37-107-6). This product mostly contains low-molecular-weight oligomers (<500 Da), i.e., dimers containing four oxygen atoms (C_18_H_18_O_4_) and trimers with six oxygen atoms (C_27_H_26_O_6_), with a weight percent ratio of C/H/O that equals 76:6:18 [49]. Lignin was fractionated by molecular sieves from mesh #60–120, and the fraction of #100–120 was used in this study (125 μm ≤ nominal size ≤ 149 μm). The hydrolytic lignin was well dissolved using a vortex stirrer followed by 30 min of sonication in an acetone/water (9:1 volume ratio) mixture. This solution was then atomized in the gas phase by a Constant Output TSI 3076 atomizer into the pyrolysis reactor (refer [1,2] for details).

### 3.2. IR Laser-Powered Homogeneous Pyrolysis (LPHP) Reactor

The general features of the LPHP reactor, which facilitate the homogeneous pyrolysis of organic compounds, are described in detail elsewhere [3,4]. The schematic of the IR LPHP reactor, which is irradiated from the side window by an IR CO_2_ laser, is represented in Figure S1. A continuous-wave cw-CO_2_ laser source was used at the P (20) line of the 00°1 → 10°0 transition (10.59 μm) with a maximum output of 40 W (Synrad Firestar CO_2_ laser, FSV40KWD, Manufacturer: Synrad, Inc., Mukilteo, WA, USA). A set of highly transparent KBr disks to the CO_2_ laser irradiation (10.6 μm) was used as an entry window for the laser reactor cell. The reactor is a Pyrex glass tube (i.d. = 20 mm, length = 10 cm) fitted from both sides with KBr windows. To avoid the destructive effect of moisture on the hygroscopic window material and the deposition of expected heavy intermediates from HL pyrolysis on the surfaces of both windows, they were protected by direct flow of N_2_ through the reaction cell close to the windows, as shown in Figure S1. The laser energy is absorbed by the sensitizer gas SF_6_, which is mixed with the initial flow of the lignin dispersed into N_2_ carrier gas and rapidly transferred to the ambient gas and reactants. An important characteristic of SF_6_ is its general non-reactivity, i.e., it does not pyrolyze significantly below 1350 °C [50]. The secondary tar-forming reactions are limited in the LPHP reactor because of the sharp temperature drop on the border of the hot volume reaction zone and the corresponding drastic decrease in the chain propagation reaction rates [3]. In other words, the extremely pronounced temperature drop from the central axis of the LPHP reactor toward the surrounding areas has a quite different effect on the various reaction steps in comparison with a conventionally heated tube reactor, as shown in a number of earlier publications [50,51,52,53,54,55,56].

#### LPHP Countercurrent Reactor

The countercurrent design reactor was placed in an isothermal reactor, as shown in Figure 1 (refer to LPHP co-current reactor in the Supplementary Materials, an inset in Figure S2); the wall temperature was maintained at 300 °C to avoid condensation/deposition on the cold walls of reactor. Most deposits were easily collected from the neck of the sampling port, and, at the same time, analysis of the condensable gases trapped at −196 °C could be undertaken. This reactor is made from Pyrex with a 20 cm length of heated wall using an electric furnace. The unique countercurrent design with various tube sizes (I.D.= 6, 11, and 20 mm), and the side injection of the N_2_ gas ensured the confinement of the reactive hot zone in the target zone. Dispersed hydrolytic lignin in the gas phase was provided by a commercially available TSI 3076 Constant Output atomizer (Shoreview, MN, USA) operated at a 3–5 L/min flowrate, and 2% SF_6_ was provided in the N2 gas mixture.

Due to the complexity of the direct temperature measurements, computational numerical modeling was undertaken to simulate and identify the temperature distribution in the reactor. COMSOL Multiphysics (v5.3a, COMSOL Inc., Burlington, MA, USA), a finite element analysis (FEA) software package, was used to solve the ordinary differential equations (ODEs) and partial differential equations (PDEs) involved in the laser light absorption and transition into heat transfer in the fluid and fluid dynamic models, using a procedure modified in our previous work [4]. The initial phase of computational temperature measurements, along with the experimental validation for a similar system, can be found elsewhere [3].

The details of the results obtained from the standard analytical techniques, namely GC-MS, EPR, FTIR, and GPC, are presented in the Supplementary Materials.

## 4. Conclusions

The fast pyrolysis of hydrolytic lignin (HL) dispersed in the gas phase was performed in an IR CO_2_ laser-powered homogeneous pyrolysis (LPHP) reactor. Lignin breaks down to fragments and forms GC-MS-detectable PAHs (MW < 300 Da). The phenolic compounds were not observed among products at higher than ~20 W non-focused laser power conditions. GPC analysis revealed a partial fragmentation of HL (at ~10–15% conversion) deposited on the cold area of the LPHP reactor. PAHs and soot-like substances were dominant components of the deposits at higher than 20–22W laser powers. The dominant fraction of lignin (~80%) transported through the hot zone consists of the decomposed neutral fragments, oligomer radicals, and non-reacted intact macromolecules of lignin.

A traditional combustion chemistry mechanism relevant to the soot formation in hydrocarbon flames at elevated temperatures was applied to explain the formation of PAHs under laser irradiation, involving the formation and reactions of CPD radicals. An alternative, “heterogenous” pathway for the generation of PAHs (parallel to the homogeneous pathways) is also proposed, involving reactions of adsorbed on HL macromolecules CPDa radicals.

Non-catalytic gas-phase deconstruction methods, such as the homogeneous pyrolysis of lignin dispersed in the gas phase, as explored in the current paper, could become more practically significant as potential biodiesel surrogates if they achieve high yields and selectivity toward low-molecular-weight PAHs.

## Figures and Tables

**Table 1:** FTIR characteristics of the analyzed samples.

Wavenumber (cm^−1^)	Peak Assignment
3394	O–H stretching in hydroxyl groups in phenolic and aliphatic structures [16,17]
2936	C–H stretching in methoxy, methyl, and methylene [16]
2846	C=O stretching in p-substituted aryl ketones [16]
1700	C=C stretching in aromatic ring (S) [16]
1596	C=C stretching in skeletal aromatic ring (S > G) [17,18]
1511	C=C stretching in skeletal aromatic ring (G > S) [17,18]
1458	C–H bending in methoxy and methylene [17]
1422	C–H in-plane deformation combined with skeletal aromatic ring [17]
1367	O–H in-plane bending [18];O–H in phenol, aliphatic C–H stretch in CH_3_ [17]
1326	C–H bending in aromatic ring (S or condensed G) [17]
1220	Aryl–O of aryl–OH and aryl–OCH_3_ [17]
1119	C–H bending in aromatic ring (S > condensed G) [17]
1085	C–O deformation in secondary alcohols and aliphatic ethers [17]
1030	C–O stretch in O–CH_3_ and C–OH [18]
915	C–H out-of-plane deformation of aromatic ring [17]
834	C–H out-of-plane in H unit and C_2,6_ of S unit [17]
726	C–H wags for an aromatic fused ring [18]

**Table 2:** The weight-average (M_w_) and number-average (M_n_) molecular weights of lignin and pyrolyzed lignin.

	HL	PHL20	PHL30	PHL40	PHL50	PHL60
M_w_ (g/mol)	5591	2298	1985	1947	1601	763
M_n_ (g/mol)	1397	614	325	309	354	113
PDI	4.0	3.7	6.1	6.3	5.1	6.7

## Data Availability

Data available upon request.

## Supplementary Materials

The following supporting information can be downloaded at: https://www.mdpi.com/article/10.3390/molecules30102215/s1, Figure S1: Schematic diagram of lignin laser-powered homogeneous pyrolysis (LPHP) flow reactor; Figure S2: FTIR data of the pyrolyzed lignin samples from LPHP reactors collected from the different locations of the reactor (labeled locations 1, 2, and 3 in the inset picture). The pyrolysis conditions were as follows: IR laser power ~22 W, main flow through atomizer 1 L/min, auxiliary flows 350 mi/min, SF6 content in main flow ~3% in (*v*/*v*); Table S1(a,b): The integral intensity of characteristic IR absorption bonds of OH (alcoholic and phenolic), C-H (stretching aliphatic), and CH_3_ (methoxy) for initial HL and pyrolyzed lignin sampled from locations 1, 2, and 3 (inset in Figure 1); The ratio of integral intensity of characteristic IR absorption bonds of OH (alcoholic and phenolic), C-H (stretching aliphatic), and CH_3_ (methoxy) toward aromatic skeletal mode in lignin (1513 cm^−1^), depending on pyrolyzed lignin sampled from locations 1, 2, and 3 (inset in Figure 1); Tables S1–S4: Compounds identification at different laser power; Figure S3: Comparison of CPD reference EPR spectrum from pyrolysis of tricarbonylcyclopentadienylmanganese (black line, reaction 1) with traces of CPD radicals detected from pyrolysis of *p*-coumaryl alcohol at elevated temperatures (>700 °C); Figure S4: Formation of substituted cyclopentadienyl (CPD) radical from decomposition of *p*-coumaryl alcohol based on M06-2X/6-31G(d,p) DFT level calculations; Figure S5: Stepwise formation of small, GC-detectable PAHs (as an example) through HACA and cyclopentadienyl radical (CPD) addition (and CH_3_ removal) in LPHP reactor; Figure S6: Pyroprobe 1000 for fast pyrolysis of hydrolytic lignin. References [57,58,59,60,61,62,63,64,65,66,67,68,69,70,71,72,73,74,75,76,77] are cited in the Supplementary Materials.
