# Supplementary material for: Laser-Powered Homogeneous Pyrolysis (LPHP) of Lignin Dispersed into Gas Phase
Source: Molecules. 2025 May 19;30(10):2215. doi: 10.3390/molecules30102215 (PMC12113869; doi:10.3390/molecules30102215)
Supplement: Supplementary file 1 [file molecules-30-02215-s001.zip › molecules-3595281-supplementary.pdf]

Supplementary Materials

Laser-Powered Homogeneous Pyrolysis (LPHP) of Lignin Dispersed into Gas Phase

Mohamad Barekati-Goudarzi [1], Lavrent Khachatryan [1], Rubik Asatryan [2,*], Dorin Boldor [3] and Bert C. Lynn [4]

[1] Department of Chemistry, Louisiana State University, Baton Rouge, Louisiana 70803, USA
[2] Department of Chemical and Biological Engineering, University at Buffalo, The State University of New York, Buffalo, NY 14260, USA
[3] Department of Biological & Agricultural Engineering, LSU, and LSU Ag. Center, Baton Rouge, Louisiana 70803, USA
[4] Department of Chemistry, University of Kentucky, Lexington, Kentucky 40506, USA

S1. IR LPHP "Wall-less" reactor

A schematic of the IR LPHP reactor in flow condition is shown in Figure S1. The reactor consisted of a Pyrex glass tube (i.d. = 20 mm, length = 10 cm) fitted from both sides with KBr windows, highly transparent to the CO_2 laser irradiation (10.6 μm). To avoid moisture's destructive effects on the hygroscopic window material and the deposition of heavy intermediates from HL pyrolysis on the surfaces of both windows, they are protected by a direct flow of N_2 through the reaction cell close to the windows, as shown in **Figure S1**.

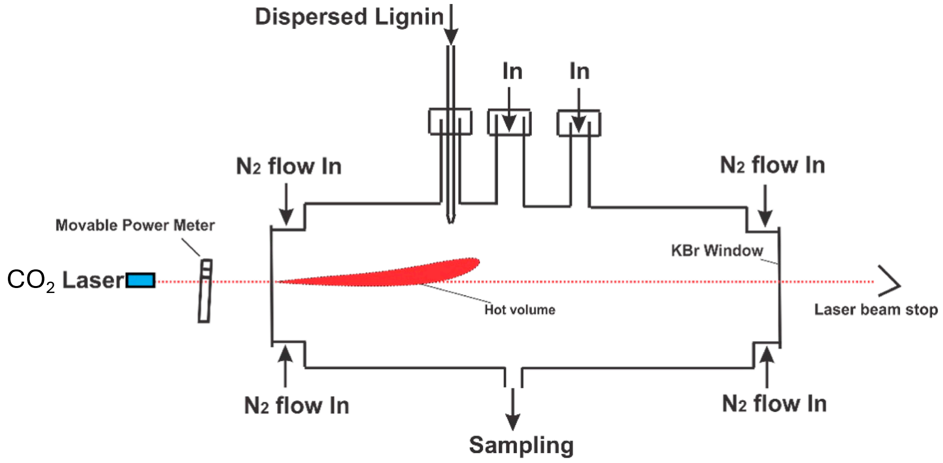

Figure S1. Schematic diagram of lignin laser-powered homogeneous pyrolysis (LPHP) flow reactor.

As a sensitizer in the gas mixture, SF_6 exhibits a relatively fast vibration–translation relaxation time—approximately 10^{-5} seconds at 1000 K and one atmosphere of total pressure[50]. The absorbed laser energy is rapidly transferred to the ambient gas containing the reactants. A key characteristic of SF_6 is its non-reactivity; it does not pyrolyze below 1350 °C [50].

S2. Experimental Analytical Methods

GC-MS Analysis

The volatile fraction of products was analyzed by a GC/MS system (Agilent 6890N/5973N GC/MSD System, Santa Clara, CA, USA) quipped with a 5973N mass selective detector-MSD) equipped with a capillary column (Agilent J&W DB-5 ms Ultra Inert GC Column, 30 m × 0.25 mm × 0.25 μm). The separation method was programmed as follows: 40 to 180°C at 4 °C/min and 1 min hold time at 180 °C, 10 °C/ min to 280 °C, and 5 min hold time at final temperature. A seven-minute delay time was used to ensure complete removal of the solvent. The MS detector was operated in the EI mode at 70 eV and with a mass detection range of 10–650 (m/z) and a frequency of 2.5 scans/s.

EPR Analysis

The EPR spectra of the products of the pyrolysis were recorded using a Bruker EMX-20/2.7 EPR spectrometer (Bruker Instruments, Billerica, MA) with dual cavities, an X-band (100 kHz), and a microwave frequency of 9.516 GHz. To obtain comparable results, the instrumental analysis parameters were chosen as follows: sweep width 200 G, EPR microwave power from 0.5 to 64 mW, modulation amplitude ≤ 4 G, and time constant and sweep time in most cases were 10.24 ms and 167.77 s, respectively. The exact g-value and the intensity of the obtained spectra for each measurement were compared with a 2,2-diphenyl-1-picrylhydrazyl (DPPH) standard to compensate for the random variations imposed by ambient conditions.

FTIR Analysis

An infrared spectrum of the initial lignin sample and pyrolysis products was recorded using an FTIR instrument (Bruker Tensor 27, Ettlingen, Germany). The transmittance spectrum was collected from 400 to 4000 cm^{-1} with a spectral resolution of 4 cm^{-1} and 16 scans using a Pike Miracle ATR cell equipped with a ZnSe single crystal.

Gel Permeation Chromatography (GPC)

In order to relatively examine the molecular weight distribution of lignin macromolecules before and after pyrolysis at various temperatures, the gel permeation chromatography (GPC) technique was performed. Prior to analysis, the hydrolytic lignin and the gas-phase pyrolysis product were subjected to acetylation according to the literature [57]. An Ultimate 3000 HPLC system (Dionex Corporation, Sunnyvale, CA) with an ultraviolet (UV) detector at a wavelength of 280 nm was used to measure the number-average molecular weight (M_n) and weight-average molecular weight (M_w). The macromolecules diffuse in different proportions through a Mixed-D PLgel column (5 μm particle size, 300 mm × 7.5 mm i.d, Polymer Laboratories, Amherst, MA) at 80 °C using a mobile phase of THF at a flow rate of 0.5 ml/min, depending on their size. The results were calibrated using low-molecular-weight polystyrene standards (product no. 48937 from Sigma-Aldrich).

S3. LPHP Co-current Reactor:

Some preliminary results from the LPHP of lignin in a non-isothermal reactor (Figure S1) were reported previously [3,4].

A modified co-current LPHP reactor is illustrated in the inset picture, as shown in Figure S2. The FTIR data of the pyrolyzed lignin samples collected from the various locations of the reactor (labeled 1, 2, and 3 on the blue background in the inset picture) are also depicted in Figure S2. Due to the uncertainty of the sample amounts analyzed by FTIR, it is difficult to compare quantitatively the initial and pyrolyzed samples. However, the trend of changes in HL macromolecule can be identified by comparing the ratios of

characteristic peaks before and after pyrolysis (summarized in Table S1 (a) integral intensity, (b) ratio of integral intensity) vs. a standard reference bond. The ratio of the integral intensity of the characteristic IR absorption bonds for OH (alcoholic and phenolic), C-H (stretching aliphatic), and CH_3 (in methoxy) toward aromatic skeletal frequency in lignin (1513cm^{-1} as a reference bond), shown in Table S1, depends strongly on the sample location (i.e., locations 1, 2, or 3, inset of Figure S2) in the reactor for the pyrolyzed lignin.

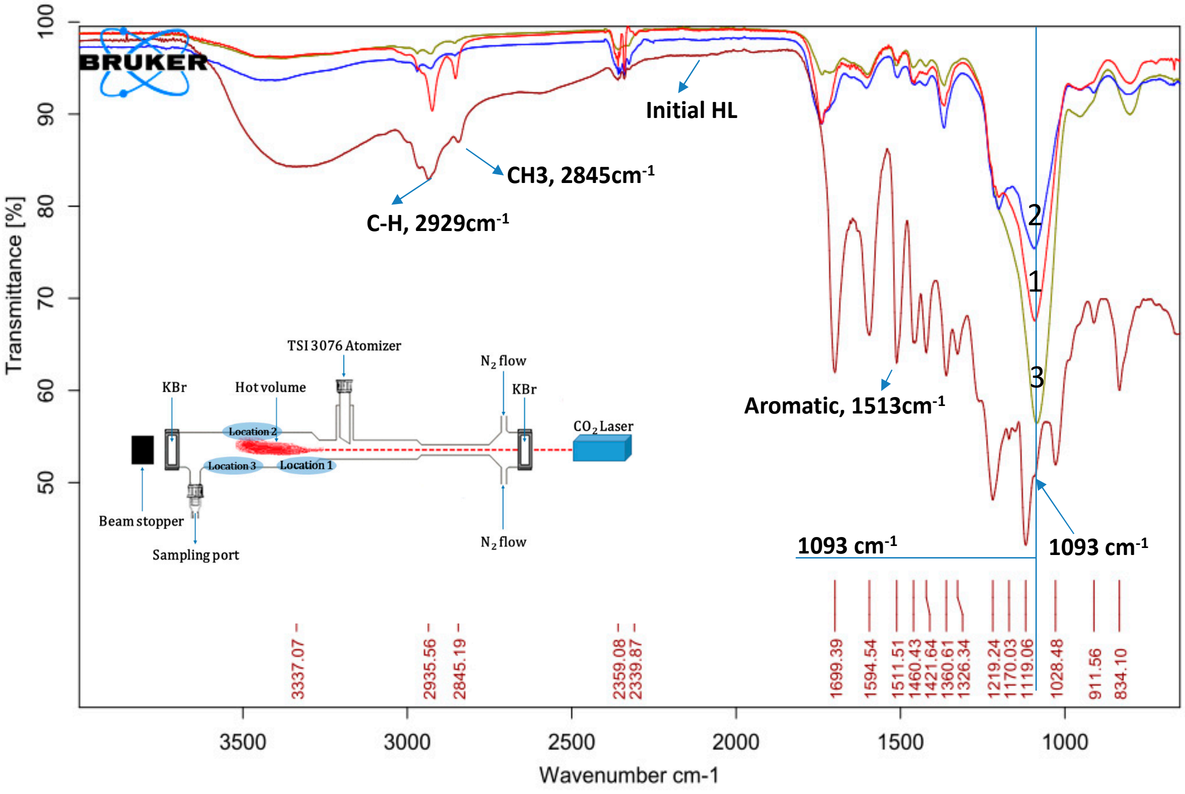

Figure S2. FTIR data of the pyrolyzed lignin samples from LPHP reactors collected from the different locations of the reactor (labeled locations 1, 2, and 3 in the inset picture). The pyrolysis conditions were as follows: IR laser power ~22W, main flow through atomizer 1L/min, auxiliary flows 350mi/min, SF6 content in main flow ~ 3% in (v/v).

Table S1. a). The integral intensity of characteristic IR absorption bonds of OH (alcoholic and phenolic), C-H (stretching aliphatic), and CH_3 (methoxy) for initial HL and pyrolyzed lignin sampled from locations 1, 2, and 3 (inset in Fig. 1).

IR wavenumber cm $^{-1}$	OH at 3337	C-H at 2929	CH_3 at 2845	Reference at 1513
Initial lignin, HL	12.184	0.097	0.151	2.794
Location 1	1.621	0.541	0.25	0.011
Location 2	4.514	0.95	0.025	0.166
Location 3	1.678	0.053	0.009	0.174

Table S1. b). The ratio of integral intensity of characteristic IR absorption bonds of OH (alcoholic and phenolic), C-H (stretching aliphatic), and CH_3 (methoxy) toward aromatic skeletal mode in lignin (1513 cm^{-1}), depending on pyrolyzed lignin sampled from locations 1, 2, and 3 (inset in Fig. 1).

IR wavenumber cm $^{-1}$	OH at 3337	C-H at 2929	CH_3 at 2845	Reference at 1513
Initial HL	4.361	0.035	0.054	1.0
Location 1	147.36	49.18	22.73	1.0
Location 2	27.19	5.72	0.15	1.0
Location 3	9.64	0.30	0.05	1.0

The ratio of the given peak area over the reference peak (at 1513 cm^{-1}) area grows significantly (Table S1 (b)), especially for **location 1,** where the laser beam first hits the flow as it enters from the middle port. The substantial difference in the ratios in comparison with the initial lignin (ref. 1st column at 3337 cm^{-1}, Table S1 (b)) evidence that the characteristic skeletal mode at 1513 cm^{-1} decreases significantly (from 2.794 to 0.011, Table S1 (a)). The same trend was observed by comparison with another reference band at 1595 cm-1 (Figure S2), which is C=C stretching in the skeletal aromatic ring (not shown), most probably by loosening of the aromaticity. All the ratios (Table S1 (b)) increase drastically due to the sharp decrease in the aromatic-related peaks' areas at 1513 cm^{-1} (Tables S1 (a) and (b)) or at 1595 cm^{-1} (not shown). Concomitantly, a peak at 1093 cm^{-1} appears to be dominant as lignin decomposes, as shown in **Figure S2**. This peak likely corresponds to the trace amounts of carbohydrates (for instance, traces of hemicellulose released from industrial manufacturing of HL) having a similar peak in the range extending from 852 to 1186 cm^{-1} [17,58,59]. Note that this peak centered at 1093 cm^{-1} is completely missing in the countercurrent configuration of the LPHP reactor (*vide infra*), likely indicating the mostly complete decomposition of this trace carbohydrate in the countercurrent reactor.

Thus, to avoid an uneven distribution of the depolymerized lignin on the walls of the co-current reactor (Figure S2 inset) and condensation reactions on the cold walls, while allowing for more of the sample to be

collected through a sampling port, the design of the LPHP reactor was updated in a counterflow configuration (**Figure 1** in the main text).

S4. Results

Table S1: Compound identification for LPHP reactor at 20% laser power

Label	Name	Formula	RT	Area
1	guaiacol	C7H8O2	13.18	4.01E+05

Table S2: Compound identification for LPHP reactor at 40% laser power

Label	Name	Formula	RT	Area
1	1H-Indene, 1-ethylidene-	C11H10	7.948	5.96E+05
2	Naphthalene, 2-methyl-	C11H10	8.28	5.26E+05
3	Naphthalene, 2-ethenyl-	C12H10	10.014	2.31E+05
4	Naphthalene, 2-ethyl-	C12H12	10.296	2.78E+05
5	Acenaphthene	C12H10	10.641	2.26E+05
6	Naphthalene, 2-ethenyl-	C12H10	11.053	4.82E+05
7	Acenaphthylene	C12H8	11.335	1.23E+06
8	Benz[a]azulene	C14H10	18.259	6.09E+05
9	Fluoranthene	C16H10	24.174	3.92E+05

Table S3: Compound identification for LPHP reactor at 60% laser power

Label	Name	Formula	RT	Area
1	1H-Indene, 1-ethylidene-	C11H10	7.739	1.67E+06
2	Naphthalene, 2-methyl-	C11H10	8.077	4.91E+06
3	Naphthalene, 2-methyl-	C11H10	8.415	3.38E+06
4	Naphthalene, 2-ethenyl-	C12H10	10.063	2.47E+06
5	Naphthalene, 1,8-dimethyl-	C12H12	10.352	1.65E+06
6	Acenaphthylene	C12H8	10.629	3.08E+06
7	Naphthalene, 2-ethenyl-	C12H10	11.219	5.24E+06
8	Acenaphthylene	C12H8	11.668	2.40E+07
9	Acenaphthene	C12H10	12.233	8.39E+05
10	Fluorene	C13H10	13.986	2.38E+06
11	1H-Phenalene	C13H10	14.447	3.86E+06
12	9H-Fluorene, 9-methyl-	C14H12	15.246	1.23E+06
13	Phenanthrene	C14H10	18.523	1.41E+07
14	Diphenylacetylene	C14H10	18.659	3.22E+06
15	4H-Cyclopenta[60]phenanthrene	C15H10	20.897	1.88E+06
16	Fluoranthene	C16H10	23.553	4.74E+06
17	Pyrene	C16H10	24.488	1.06E+07

Table S4: Compound identification for LPHP reactor deposits on the outlet

Label	Name	Formula	RT	Area
1	Fluoranthene	C16H10	23.356	2.59E+05
2	Pyrene	C16H10	23.781	1.70E+05
3	Pyrene	C16H10	24.174	9.28E+05

4	Pyrene, 1-methyl-	C17H12	26.646	2.87E+05
5	Cyclopentapyrene	C18H10	28.656	4.91E+05
6	Naphthacene-	C18H12	28.81	1.87E+05
7	Cyclopentapyrene	C18H10	29.511	1.45E+06
8	Naphthacene, 5,12-dihydro-	C18H14	29.696	3.16E+05
9	Benzofluoranthene	C18H10	29.825	3.00E+05
10	9H-Cyclopenta-pyrene	C19H12	31.958	5.75E+05
11	9H-Cyclopenta-pyrene	C19H12	32.216	2.29E+05
12	Benzo(e)pyrene, 9,10-dihydro-	C20H14	34.166	6.51E+05
13	Anthracene, 9-phenyl-	C20H14	35.033	2.52E+05
14	1,2'-Binaphthalene	C20H14	35.235	7.04E+05
15-18	Not identified			

S5. Alternative Mechanisms for Formation of PAHs And Soot-Like Substances

The HACA mechanism is a basic approach to soot formation widely discussed in the literature [5,28,43,61]. Some alternative pathways have also been recently proposed for the coalescence of PAHs and the formation of the first soot nuclei particle, *soot inception*, such as repetitive HAMA (i.e., hydrogen abstraction methyl addition) [62], and CAHM (carbon addition hydrogen migration) [25].

The purely gas-phase molecular growth model associated with the HACA mechanism, which was initially thought to result in particle nucleation, was shown to be too slow when considered on the time scale of the soot particle inception [63,64]. Hence, *PAH dimerization* has been put forward as the initial step in soot nucleation (reactive dimerization of small aromatics [75]). The electrostatic and dispersive forces were suggested to be responsible for such processes, even at elevated (flame) temperatures [66], which, however, can be ruled out based on the boiling/sublimation temperatures of larger PAHs; instead of condensation, the small aromatics would rather evaporate at those temperatures. [23]

To summarize, below are the pathways for the formation of the first aromatic ring suggested in the literature:

Even-carbon-atom pathways that involve the addition of acetylene to n-C_4H_3 or n-C_4H_5 radicals:

$C_4H_5 + C_2H_2 \rightarrow C_6H_6 + H$ (S1)

Odd-carbon-atom pathways via the recombination of propargyl radicals (C_3H_3), reaction 2 or a combination of two cyclopentadienyl (CPD) radicals, reaction 3 (or a CPD and a methyl radical-not shown):

$C_3H_3 + C_3H_3 \rightarrow C_6H_6$ (S2) or

$2\ C_5H_5 \rightarrow C_{10}H_8 + 2H$ (S3)

A combined version involving reactions between propargyl and acetylene to form a cyclopentadienyl (CPD) radical [67], or between cyclopentadienyl and acetylene to produce C6 through C7 intermediates [68].

Many questions remain with respect to the homogeneous soot formation pathways [67,69,70]. The role of the resonance-stabilized cyclopentadienyl and other aromatic radicals in soot formation has been indirectly demonstrated in several studies. [75,76] Note that GC-MS-detectable 4-ring PAHs (Figure 3), such as pyrene and fluoranthene, can also serve as precursors for the formation of soot particulates. To confirm the formation of resonantly stabilized benzyl ($C_6H_5CH_2$•) and indenyl (•C_9H_7) radicals in the reaction environment, these active species may produce the mentioned PAHs according to the theoretical calculations [71].

In a recent breakthrough study, Johansson et al. [72] suggested that the resonance-stabilized radicals (RSRs) may explain soot inception and growth in high-temperature flames through step-by-step clustering of the hydrocarbons via a radical chain reaction (CHRCR) mechanism.

CPD radicals from pyrolysis of p-CMA (p-coumaryl alcohol) [38].

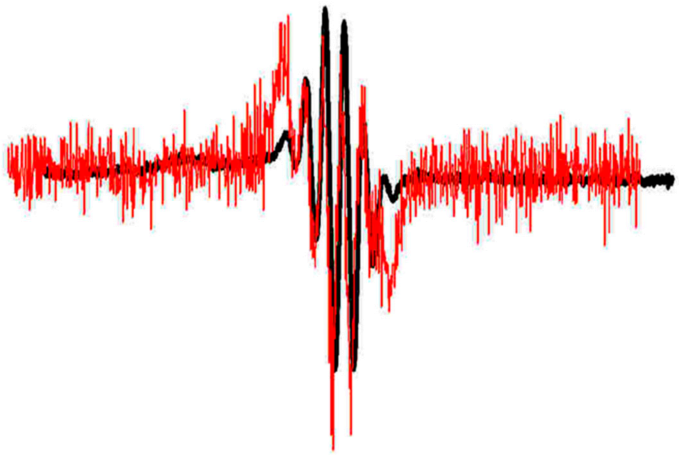

Figure S3. Comparison of CPD reference EPR spectrum from pyrolysis of tricarbonylcyclopentadienylmanganese (black line, reaction 1) with traces of CPD radicals detected from pyrolysis of p-coumaryl alcohol at elevated temperatures (>700 °C).

$$(C_5H_5)Mn(CO)_3 \rightarrow C_5H_5 + Mn + 3CO$$

reaction (S1)

S6. Formation of derivatized PAHs in LPHP reactor[42].

The high temperature in a narrow hot zone under laser irradiation (V_{eff}/V_o ~ 10^{-1} to 10^{-3} depending on the laser power, where V_{eff} is the hot zone volume, and Vo is the the reactor volume), which can reach 1000 oC and higher temperatures (**Figure 2** in the main text), promotes the removal (stripping) of the most of functional groups from lignin macromolecules, intermediate oligomers, and radicals. PAHs containing side-chain hydrocarbons can also be formed**.** CPD radicals are known to play key roles in the PAH formation, serving as one of the critical precursor radicals [42].

Figure S4 provides a theoretical explanation of the formation of a derivative CPD radical from the pyrolysis of one of the three main lignin monomers/precursors and a model compound, *p*-coumaryl alcohol (*p*-CMA) [42].

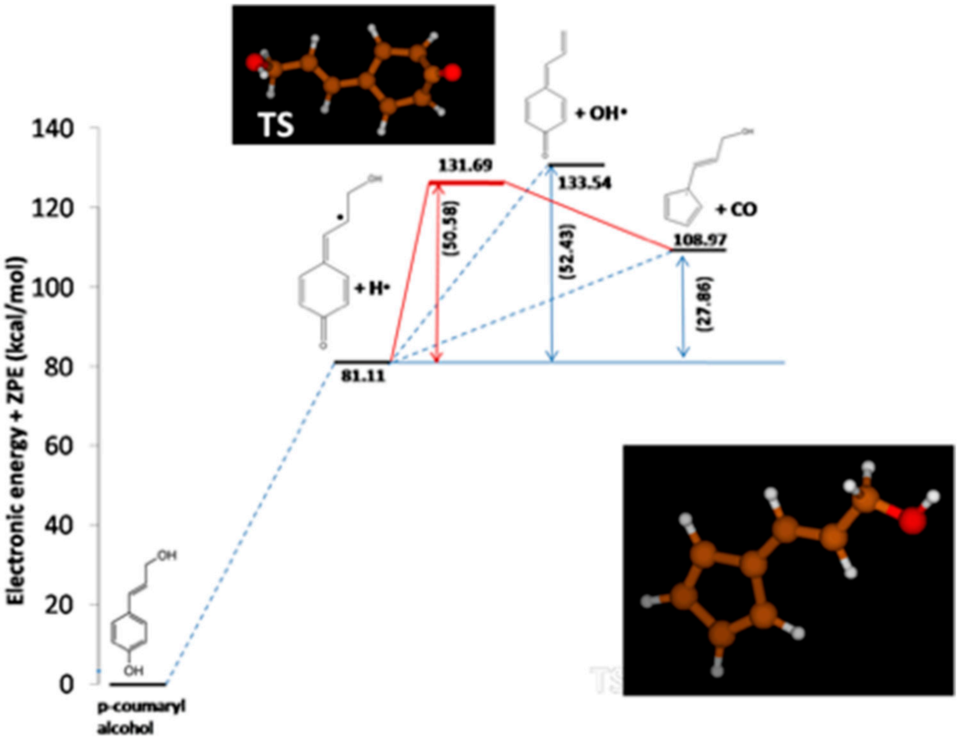

Figure S4. Formation of substituted cyclopentadienyl (CPD) radical from decomposition of *p*-coumaryl alcohol based on M06-2X/6-31G(d,p) DFT level calculations.

The expulsion of CO to form 3-hydroxy propen-1 CPD radical in the gas phase faces an activation barrier of only 51 kcal/mol calculated at M06-2X/6-31G(d,p) level of theory (**Figure S4**), which can be well surmounted at elevated temperatures[73] in the laser beam. Further dimerization of the substituted CPD radical may result in the formation of derivatized PAHs (not shown) analogous to the process illustrated in **Figure S5** below for pure CPD radicals.

Formation of Naphthalene and small PAHs

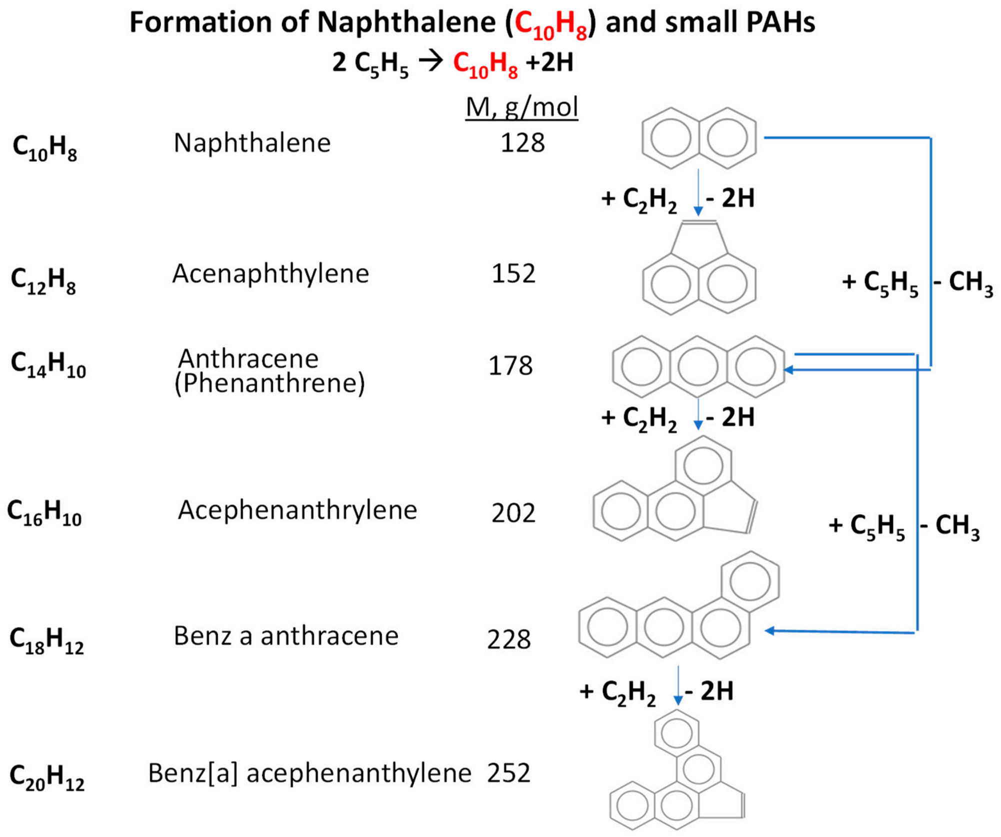

Figure S5. Stepwise formation of small, GC-detectable PAHs (as an example) through HACA and cyclopentadienyl radical (CPD) addition (and CH_3 removal) in LPHP reactor.

S7. CPD (cyclopentadienyl) radicals "in motion":

A mobility phenomenon of the adsorbed species, particularly CPD radicals (CPDa), on the surfaces is known from recent [74] and early publications [75–77] Thermal reactions of phenol on metal surfaces, Pt(111), including reaction intermediates between -125 ºC and 827 ºC, have been reported [74]. During heating, the O-H bond breaks at temperatures as low as -73 ºC. The product of a phenolic O-H bond dissociation, the phenoxy moiety, has an electron-delocalized quinoidal structure (a conjugated structure that is prone to form char). However, a different stoichiometry of the decomposition of phenoxy can also occur, such as

$C_6H_5O(a) \rightarrow CO(g) + H_2(g) + (C_5H_3(a))$, (S4)

where (a) stands for adsorbed moieties, and (g) stands for gases.

(C_5H_3(a)) indicates an average stoichiometry, not necessarily an adsorbed species. (C_5H_3(a)) may generate propargyl radical (C_3H_3 (a) +C_2(a)), which is an important intermediate radical to produce the key first ring intermediate: benzene [74]. On the other hand, H_2(g) + (C_5H_3(a)) can also

reproduce the CPDa radical. The CPDa radical, C_5H_5a, leads to the formation of acetylene and propargyl radical (key moieties responsible for soot inception in hydrocarbon flames, Section 3.4.1), rxn (5):

$C_5H_5a \rightarrow C_2H_2a$ (or gas phase) + C_3H_3a (S5)

The behavior of CPD radicals stabilized on metal surfaces has also been reported in early publications. [75–77]. Condensed film of cyclopentadiene (c-C_5H_6) was deposited onto the Rh surface at -193 ºC in the chamber at 10^{-9} torr [75]. The formation of oligomers was observed at low temperatures (-193 ºC – -173 ºC) following irradiation with vacuum UV photons. The dehydrogenation of c-C_5H_6 starts at T >-123 ºC by the formation of adsorbed C_5H_5 species identified by UV photoelectron spectroscopy. Fragment species like C_5H_5 (Fe) were strongly adsorbed after the desorption of ferrocene $Fe(C_5H_5)_2$ from the Ag(100) surface in a high-vacuum chamber (partial dissociation of ferrocene occurred under UV light) [76]. The thermal desorption peak occurred at ~ -23 ºC for both fragments (for Fe- 56amu and C_5H_5 – 65amu). Even at room temperature, the thermal mobility of chemisorbed radicals, C_5H_5, on Ag(100) has been detected by STM [77]. The phenomenon called "**molecules in motion**" is non-destructive; in fact, no fragmentation of the radical C_5H_5 has been observed at low temperatures. [77]

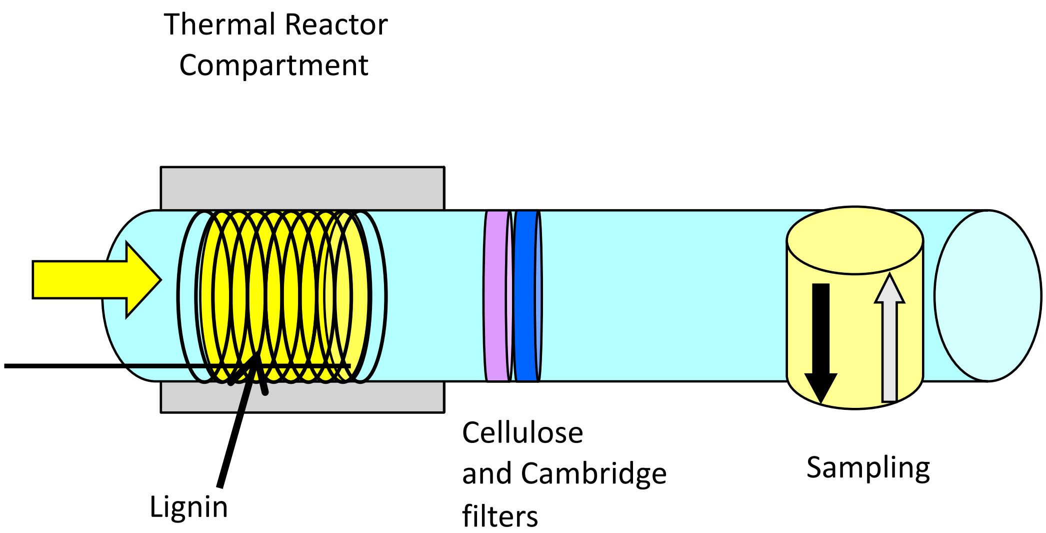

Figure S6. Pyroprobe 1000 for fast pyrolysis of hydrolytic lignin.

S8. Lignin Pyrolysis in Pyroprobe [46]

Lignin samples were pyrolyzed in a commercial Pyroprobe 1000 (CDC Analytical. Inc) using a continuous flow system, Figure S6. The pyrolysis parameters were as follows:

Lignin downloaded ~ 80 mg in a quartz reaction tube (I.D. = 6 mm);

Heating rate 100 ˚C/s;

Retention time (RT) 0.05 – 0.1 sec at flow rate 100–200 cm^3/min.

References

3. Khachatryan, L.; Barekati-Goudarzi, M.; Kekejian, D.; Aguilar, G.; Asatryan, R.; Stanley, G.G.; Boldor, D. Pyrolysis of Lignin in Gas-Phase Isothermal and cw-CO2 Laser Powered Non-Isothermal Reactors, *Energy & Fuels* **2018**, *32*, 12597–12606

4. Kekejian, D.; Khachatryan, L.; Barekati-Goudarzi, M.; Boldor., D. Implication of COMSOL to Laser Powered Non-Isothermal Reactors for Pyrolysis in the Gas Phase. *COMSOL conference* **2018**, Boston Marriott Newton, October 3–5

5. Zhou, H.; Wu, C.; Onwudili, J.A.; Meng, A.; Zhang, Y.; Williams, P.T. Polycyclic Aromatic Hydrocarbon Formation from the Pyrolysis/Gasification of Lignin at Different Reaction Conditions, *Energ. Fuels* **2014**, *28*, 6371–6379

17. Faix, O. Classification of Lignins from Different Botanical Origins by FT-IR Spectroscopy, *Holzforschung* **1991**, *45*, 21. https://doi.org/10.1515/hfsg.1991.45.s1.21.

23. Wang, H. Formation of nascent soot and other condensed-phase materials in flames, *P. Combust. Inst.* **2011**, *33*, 41–67

25. Frenklach, M.; Singh, R.I.; Mebel, A.M. On the low-temperature limit of HACA. *P. Combust. Inst.* **2019**, *37*, 969–976

28. Mastral, A.M.; Callen, M.S. A review on polycyclic aromatic hydrocarbon (PAH) emissions from energy generation, *Environ. Sci. Technol.* **2000**, *34*, 3051–3057

32. Lu, M.; Mulholland, J.A. Aromatic hydrocarbon growth from indene, *Chemosphere* **2001**, *42*, 623

33. Yang, B.; Hu, B.; Koylu, U.O. Mean soot volume fractions in turbulent hydrocarbon flames: A comparison of sampling and laser measurements, *Combust. Sci.* **2005**, *177*, 1603–1626

38. Xu, M.X.; Khachatryan, L.; Baev, A.; Asatryan, R. Radicals from the Gas-Phase Pyrolysis of Lignin Model Compounds. p-Coumaryl Alcohol, *RSC Adv.* **2016**, *6*, 62399–62405

42. Asatryan, R.; Bennadji, H.; Bozzelli, J.W.; Ruckenstein, E.; Khachatryan, L. Molecular Products and Fundamentally Based Reaction Pathways in the Gas-Phase Pyrolysis of the Lignin Model Compound p-Coumaryl Alcohol, *J. Phys. Chem. A* **2017**, *121*, 3352–3371

43. Shukla, B.; Koshi, M. A novel route for PAH growth in HACA based mechanisms, *Combust Flame* **2012**, *159*, 3589–3596

46. Khachatryan, L.; Mascos, Z.; Dellinger, B. The Tar and Tar Radicals from Lignin Pyrolysis. *SFRBM (Society for Free Radical Biology and Medicine)* **2014**, November 19–23

50. Shaub, W.M.; Bauer, S.H. Laser-Powered Homogeneous Pyrolysis. *Int. J. Chem. Kinet.* **1975**, *7*, 509–529.

57. Liu, E.; Das, L.; Zhao, B.; Crocker; M.; Shi. Impact of Dilute Sulfuric Acid, Ammonium Hydroxide, and Ionic Liquid Pretreatments on the Fractionation and Characterization of Engineered Switchgrass, *J. BioEnergy Research* **2017**, *10*, 1079–1093.

58. Faix, O. Investigation of Lignin Polymer Models (Dhps) by Ftir Spectroscopy *Holzforschung* **1986**, *40*, 273–280

59. Yang, H.P.; Yan, R.; Chen, H.P.; Lee, D.H.; Zheng, C.G. Characteristics of hemicellulose, cellulose and lignin pyrolysis, *Fuel* **2007**, *86*, 1781–1788

60. Clapp, R.; deFur, P.; Silbergeld, E.; Washburn, P. EPA on the right track, *Environ. Sci. Technol.* **1995**, *29*, 29–30

61. Kislov, V.; Islamova, N.; Kolker, A.; Lin, S.; Mebel, A. Hydrogen Abstraction Acetylene Addition and Diels−Alder Mechanisms of PAH Formation: A Detailed Study Using First Principles Calculations, *J. Chem. Theory Comput.* **2005**, *1*, 5, 908–924.

62. Hansen, N.; Schenk, M.; Moshammer, K.; Kohse-Hoinghaus, K. Investigating repetitive reaction pathways for the formation of polycyclic aromatic hydrocarbons in combustion processes, *Combust. Flame* **2017**, *180*, 250–261
63. Frenklach, M. Reaction mechanism of soot formation in flames, *Phys.Chem.Chem.Phys.* **2002**, *4*, 2028–2037
64. Frenklach, M.; Schuetz, C.A.; Ping, J. Migration mechanism of aromatic-edge growth, *30th Inern. Symp. on Combustion, Chicago* **2004**, *Pittsburgh, The Combustion institute*, 1389–1396
65. Kholghy, M.R.; Eaves, N.A.; Veshkini, A.; Thomson, M.J. The role of reactive PAH dimerization in reducing soot nucleation reversibility. *P. Combust. Inst.* **2019**, *37*, 1003–1011
66. Herdman, J.D.; Miller, J.H. Intermolecular potential calculations for polynuclear aromatic hydrocarbon clusters. *J. Phys. Chem. A* **2008**, *112*, 6249–6256
67. Whitesides, R.; Kollias, A.C.; Domin, D.; Lester, W.A.; Frenklach, M. Graphene layer growth: Collision of migrating five-member rings. *31st Intern. Symp. on Combustion, University of Heidelberg, Germany,* **2006**, *Abstracts of Symposium Papers*, 64
68. Cavallotti, C.; Mancarella, S.; Rota, R.; Carra, S. Conversion of C5 into C6 cyclic species through the formation of C7 intermediates. *J. Phys. Chem. A* **2007**, *111*, 3959–3969. https://doi.org/10.1021/jp067117f.
69. Agafonov, G.L.; Naydenova, I.; Vlasov, P.A.; Warnatz, J. Detailed Kinetic Modeling of Soot Formation in Shok Tube Pyrolysis and Oxidation of Toluene and N-Heptane, *P. Combust. Inst.* **2007**, *31*, 575–583.
70. Violi, A.; Izvekov, S. Soot Primary Particle Formation from Multiscale Coarse-Grained Molecular Dynamics Simulation. *Proceedings of the Combustion Institute. 2007, 31, 1, 529-537. Doi:10.1016/j.proci.2006.07.240.*
71. Sinha, S.; Rahman, R.K.; Raj, A. On the role of resonantly stabilized radicals in polycyclic aromatic hydrocarbon (PAH) formation: Pyrene and fluoranthene formation from benzyl-indenyl addition, *Phys. Chem. Chem. Phys.* **2017**, *19*, 19262–19278
72. Johansson, K.O.; Head-Gordon, M.P.; Schrader, P.E.; Wilson, K.R.; Michelsen, H.A. Resonance-stabilized hydrocarbon-radical chain reactions may explain soot inception and growth. *Science* **2018**, *361*, 997–1000
73. Marinov, N.M.; Pitz, M.J.; Westbrook, C.K.; Vincitore, A.M.; Castaldy, M.J.; Senkan, S.M. Aromatic and polycyclic aromatic hydrocarbon formation in a laminar premixed n-butane flame. *Combust. Flame.* ,**1998**, *114*, 192
74. Ihm. H.; White, J.M. Stepwise Dissociation of Thermally Activated Phenol on Pt(III), *J. Phys. Chem. B* **2000**, *104*, 6202–6211.
75. Netzer, F.P. Low-Temperature Polymerization of Condensed Cyclopentadiene Induced by Uv Irradiation, *Chem. Phys. Lett.* **1988**, *146*, 566–569, 10.1016/0009-2614(88)87503-1.
76. Welipitiya, D.; Dowben, P.A.; Zhang, J.D.; Pai, W.W.; Wendelken, J.F. The adsorption and desorption of ferrocene on Ag(100), *Surf. Sci.* **1996**, *367*, 20–32,
77. Pai, W.W.; Zhang, Z.Y.; Zhang, J.D.; Wendelken, J.F. Direct visualization in manipulation of stable molecular radicals at room temperature, *Surf. Sci.* **1997**, *393*, L106–L112
